# Effects of light spectrum on the morphophysiology and gene expression of lateral branching in Pepino (*Solanum muricatum*)

**DOI:** 10.3389/fpls.2022.1012086

**Published:** 2022-09-23

**Authors:** Cheng Si, Shipeng Yang, Xiangyun Lou, Guangnan Zhang, Qiwen Zhong

**Affiliations:** ^1^Qinghai Key Laboratory of Vegetable Genetics and Physiology, Agriculture and Forestry Sciences Institute of Qinghai University, Xining, China; ^2^Qinghai University, Xining, China; ^3^College of Life Sciences, Northwest A&F University, Xining, China; ^4^Laboratory for Research and Utilization of Germplasm Resources in Qinghai Tibet Plateau, Xi’an, China

**Keywords:** Pepino, LED lamp, lateral branch, photomorphogenesis, gene expression

## Abstract

In the present study, we determined the morphological and physiological indicators of Pepino to elucidate its lateral branching responses to different light qualities using a full-spectrum lamp (F) as the control and eight different light ratios using blue light (B) and red light (R). In addition, correlation analysis revealed that the gene expression patterns correlated with lateral branching under various light treatments. Compared with the F treatment, the R treatment increased the plant height and inhibited the elongation of lateral branches, in contrast with the B treatment. The number of lateral branches did not change significantly under different light quality treatments. Moreover, correlation analysis showed that the ratio of blue light was significantly positively correlated with the length of lateral branches and significantly negatively correlated with plant height, aboveground dry weight, and other indicators. We conducted transcriptome sequencing of the sites of lateral branching at three periods under different light quality treatments. The gene related to photodynamic response, cryptochrome (*CRY*), was the most highly expressed under B treatment, negatively regulated lateral branch length, and positively correlated with plant height. Branched 1, a lateral branch regulation gene, was upregulated under R treatment and inhibited branching. Overall, the red light facilitated internode elongation, leaf area expansion, plant dry weight increase, and inhibition of lateral branching. Soluble sugar content increased, and the lateral branches elongated under blue light. Different light qualities regulated lateral branching by mediating different pathways involving strigolactones and *CRY*. Our findings laid a foundation for further clarifying the response mechanism of Pepino seedlings to light and provided a theoretical reference for elucidating the regulation of different light qualities on the lateral branching of Pepino.

## 1 Introduction

Light is one of the main factors affecting plant growth and development (Yadav et al., 2020). Plant development and structure constantly adapt to the changes in the natural light environment. Plants maximize the use of light by regulating their growth and development to respond to the surrounding light environment (Gregory, 2018). To utilize light as an important energy source, plants have evolved various signal transduction mechanisms to respond to numerous wavelengths of light in the visible spectrum, including red and blue light (Rosado et al., 2022). Red and blue light maximizes photosynthetic performance and is therefore essential for normal plant growth and development (Kharshiing and Sinha, 2016). In addition, most plants can regulate shade avoidance response by sensing blue and red wavelengths (Xie et al., 2022; Zeidler, 2022). Therefore, blue and red light is essential for plant photomorphogenesis and photosynthetic traits. Light-emitting devices (LEDs) are widely used in modern agriculture to promote the growth and development of plants along with the yield and quality (Arcel et al., 2021). Plant factory production and greenhouse light supplementation have become key technologies in protected horticulture (Yang et al., 2022).

Plant architecture is dominated by branching, which is vastly influenced by light (Liu et al., 2022). The competition between lateral branches and main branches for water, nutrients, and light hinders tomato productivity (Han et al., 2017). Blue light inhibits hypocotyl elongation in seedlings, whereas red light induces it. Cryptochrome 1 (*CRY1*) gene mediates the response to blue light to modulate the regulation of root growth and lateral branching (Zlotorynski, 2016); however, the number of lateral branches of *CRY* mutant plants is considerably reduced (Zhong et al., 2021). In addition, *CRY1* can mediate plant hormone action and promote photomorphogenic signals through 3-indoleacetic acid (IAA) and brassinosteroids (BR) (Xia et al., 2021). The inhibition of hypocotyl elongation by strigolactone depends on *CRY* and plant pigment signaling pathways (Jia et al., 2014). Strigolactone ultimately acts on *BRC1*, which is highly expressed to slow down axillary bud development (Martín-Trillo et al., 2011). The *AtBRC1* and *AtBRC2* mutants arrest axillary bud development; however, the *brc2* mutant phenotype is weaker than the *brc1* mutant (Aguilar-Martínez et al., 2007). In addition to blue light, red light plays an important role in regulating plant architecture. Phytochrome interacting factor (*PIF*) directly interacts with plant pigments to regulate the red/far-red light signaling pathway and with *CRY* to regulate blue light signaling (Huai et al., 2018; Wang P. et al., 2022). Red light inhibits hypocotyl elongation and increases hypocotyl diameter in soybeans (Wang C. et al., 2022). Photosynthesis determines crop yield and quality, depending on the concentration of Chl a, Chl b, and carotenoids, which directly affect photosynthetic capacity (Kim and Son, 2022). In the seedlings of Pepino (*Solanum muricatum*), red light inhibits plant height, leaf development, and photosynthetic characteristics, and the contents of Chl a, Chl b, and carotenoids increase with an increased ratio of red light but decrease considerably with an increased ratio of blue light (Di et al., 2021). In addition, different light can trigger different pathways of the apical dominant regulatory network. Moreover, the competition for sugar caused by the growing apical sugar sink may deprive the sugar in the lateral buds (Schneider et al., 2019).

Pepino originates from the Andes in South America and belongs to the genus Solanum (*Solanum* L.) in the Solanaceae family. Its lateral branches are clustered and germinate vigorously, which is closely related to relative species such as tomatoes and potatoes in phylogeny (Kumar et al., 2017). Wild-type tomato plants develop lateral shoots only after floral transition, and their growth follows an apical–basal pattern (Chaabouni et al., 2009), similar to Pepino. To the best of our knowledge, the biological characteristics of easy germination of lateral branches of Pepino restrict the development of the Pepino industry. Excessive growth of lateral branches disperses the nutrient supply of plants, which impedes fruiting and fruit expansion. Only a few studies have reported the model for regulating plant lateral branch development using different light qualities. The photomorphogenesis of plants is induced by light through multiple photoreceptors and abundant related plant morpho-physiological and gene expression processes (Srivastava et al., 2022). The regulatory mechanism of different light qualities on crop morphogenesis is of great significance. Controlling seedling growth by regulating light quality can help obtain robust crops while shortening the seedling cycle, providing a consulting and reference value for using light to regulate crop morphogenesis.

In this study, we used the Pepino and determined the related morphological indexes at different periods using different light quality treatments. Further, we determined the physiological indexes in response to photosynthetic characteristics to identify the dynamic response process of Pepino to physiological levels under different light quality treatments. We used transcriptome sequencing to identify the important functional genes involved in the response of lateral branching sites to light regulation. Moreover, we investigated the pathway of strigolactone hormone for the response of lateral branching to clarify the mechanism of the molecular regulation of light quality. Our findings laid a foundation for clarifying the light quality response mechanism of Pepino and provided a theoretical reference for improving the plant type of Pepino.

## 2 Materials and methods

### 2.1 Test materials and treatments

**Figure 1:**
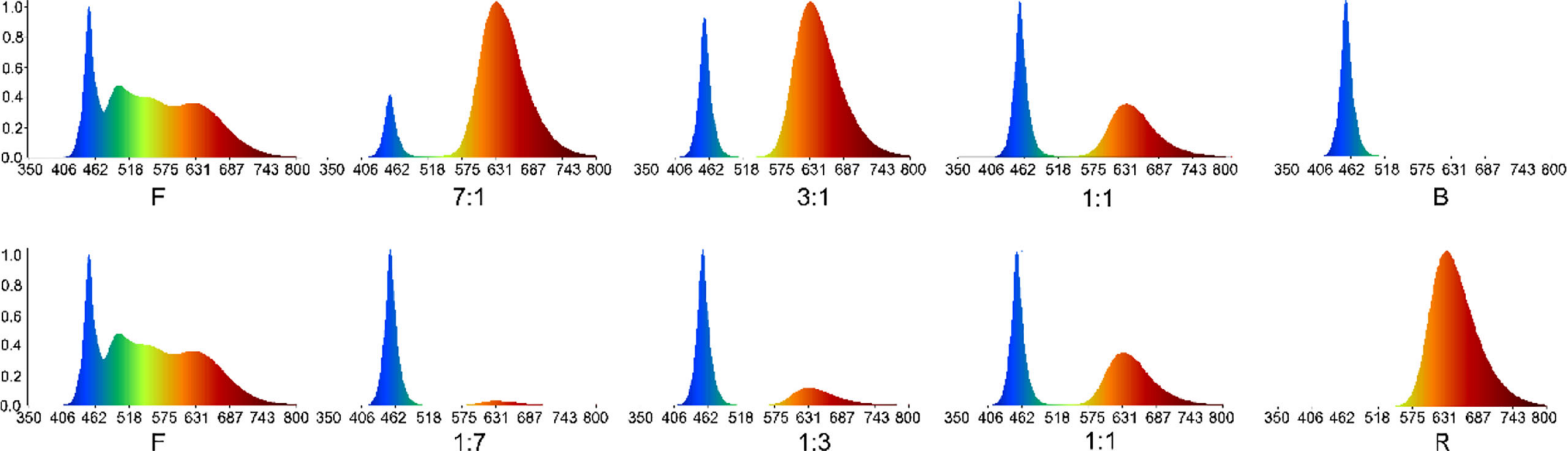
Ratios of spectral values measured under different light quality.

We used the Pepino SRF (sweet round fruit) cultivar as the plant material, provided by the Institute of Horticulture, Qinghai Academy of Agriculture Forestry Science (E 101°45′08″, N 36°43′32″). Lateral branches of 5–10 cm in length from the same position and growth vigor were selected from virus-free plants after 90 d of growth for cutting. The LED strip lights were provided by Zhejiang Little Sun Agricultural High-tech Co., Ltd. The light quality was tested in a phytotron. The environment was controlled at 25 ± 1 °C and 17 ± 1 °C during the day and night, respectively, with 60% humidity. The seedling tray was placed on a three-layer culture shelf of 120 cm in length, 50 cm in width, and 190 cm in height. Each layer of the culture holder was set up with red and blue LED lights. Full spectrum lamps (F) were used, and the light-to-mass ratio was derived through spectrometry (Hernández and Kubota, 2016), adjusting the light intensity to 100 ± 10 μMol m^–2^ s^–1^. The illumination time was 14 h/d. Indexes related to seedling phenotype were measured on days 10, 20, and 30 after treatment, and each treatment was repeated 10 times. Different light quality treatments were divided into three treatment groups: F treatment (Full spectrum) group was used as control, red-light treatment group (R-all), including LED red: blue = 1:1; 3:1; 7:1 (RB 1:1; RB 3:1; RB 7:1) and LED red (R). Blue-light treatment group(B-all), including LED red: blue = 1:1; 1:3; 1:7 (RB 1:1; RB 1:3; RB 1:7), and LED blue (B) (**Figure 1**).

### 2.2 Experimental method

#### 2.2.1 Determination of morphological indices

1. In each treatment, 10 seedlings of Pepino were randomly selected for sampling. The plant height was measured from the stem base to the growing point using a ruler with an accuracy of 0.01 cm. The diameter of the upper, middle and lower parts of the stem was measured using a Vernier caliper, and their average value was measured with an accuracy of 0.01 mm. The internode and petiole lengths were measured using a ruler with an accuracy of 0.01 cm.
2. Statistics of the lateral branch length: The node of the first true leaf was selected as the starting node for lateral bud statistics, and the lateral bud lengths of 4–6 nodes were counted in an upward direction and referred to as lateral buds at 1–6 nodes, respectively. The lateral buds of <1 mm were regarded as no lateral buds, and those of >1 mm were regarded as the effective length of lateral buds. There were five biological replicates per treatment.
3. Number of lateral buds: We counted the total number of the effective length of lateral buds.
4. Fresh weight and dry weight of plants: Five seedlings of Pepino were randomly selected for sampling in each treatment. The plants were removed from the plug, washed and dried with absorbent paper, divided into aboveground and underground parts, weighed using a balance for fresh weight with an accuracy of 0.001 g, heated at 105°C, dried at 70°C for 24 h, and weighed using a balance for dry weight with an accuracy of 0.001 g.
5. Leaf area measurement: The plant leaves were spread on a high-definition scanner (GXY-A; Zhejiang Topu Yunnong Technology Co., Ltd., Hangzhou, China) for scanning. The shaded parts were analyzed using Adobe Photoshop (v2019), and the leaf area was calculated with an accuracy of 0.001 cm^2^. Then we heated the leaves at 105°C, oven dried them at 70°C for 24 h, weighed the dry mass using a balance, and calculated the leaf mass per area (LMA) (g/cm^2^) = leaf dry mass/leaf area.

#### Determination of physiological indexes

1. Soluble sugar: After drying and crushing the fresh sample, we took 0.1 g of it into a test tube, added 10 mL distilled water, sealed it with a plastic film, extracted it twice in a boiling water bath(100°C) for 30 min, filtered the extracted solution into a 50 mL volumetric flask, rinsed the test tube and the residue repeatedly, and fixed the volume to the scale. Then, we added 2 mL of the sample extract solution into a 20 mL graduated test tube and added 0.5 mL anthrone-ethyl acetate reagent and 5 mL concentrated sulfuric acid. After full oscillation, the test tube was immediately put into a boiling water bath, maintained the temperature for 1 min, cooled to room temperature, and measured OD630 with a spectrophotometer. Soluble sugar content (%) = [X × (VT/VS) × n]/(W × 10 ^ 6) × 100%. X: The regression equation for sugar content (Regression equation: y = 0.0091x + 0.0069; R^2^ = 0.9983); VS: Volume of aspirated sample liquid; VT: Volume of extracted liquid; n: Dilution times; W: Sample dry weight.
2. Activities of sucrose-metabolizing enzymes: The fourth fully expanded leaf from the top of the plant was selected to determine the activities of sucrose metabolism-related enzymes (sucrose synthetase [SS] and sucrose phosphate synthase [SPS]). Solarbio kits (BC0580 and BC0600; Solarbio, Beijing, China) were used to determine the two enzymes.
3. Content of photosynthetic pigment: We selected fresh leaves, removed the midrib, wiped and cut them into pieces, weighed 0.2 g, and put them into a test tube. Then, we added 5 mL of 95% ethanol to soak the leaves and placed them in the dark for 24 h. Absorbance was measured, the extracted chlorophyll solution was shaken and poured into a cuvette, and 95% ethanol was used as a blank control to determine the light absorption values at 665, 649, and 470 nm. Chl a (mg·L^–1^) = 13.95A665–6.88A649; Chl b (mg·L^–1^) = 24.96A649–7.32A665; Chl (a+b) = Chl a + Chl b; carotenoids (mg·L^–1^) = (1000A470–2.05Chla–114Chlb)/245. A665、A649、A470:absorption values at 665, 649, and 470 nm.

#### 2.2.3 Expression analysis of genes related to lateral branching

Total RNA was extracted using TaKaRa MiniBEST Plant RNA Extraction Kit (TaKaRa, Beijing, China) from the bud base of Pepino seedlings treated with different light qualities (Same as 1.1) for 10, 20, and 30 d. A NanoDrop 2000 spectrophotometer (Thermo Scientific, Pittsburgh, PA, USA) was used to assess the RNA samples’ purity, concentration, and integrity. We preprocessed the downstream data to remove joints-containing and Ploy-N-containing data while filtering out low-quality data to obtain clean reads. Then, we assembled the transcripts with reference to the Pepino genome. After the assembly, the GOseq R package software was used for Gene Ontology (GO) enrichment analysis. Meanwhile, the Kyoto Encyclopedia of Genes and Genomes (KEGG) signal pathway enrichment analysis was performed using KOBAS software. Gene expression abundance was measured by fragments per kilobase of transcript per million fragments mapped (FPKM) value. Transcriptome sequencing was completed by Guangzhou Gene Denovo Biotechnology Co., Ltd. (Guangzhou, China).

### 2.3 Data processing and analysis

Microsoft Excel was used to segregate the relevant physiological data and tables. Barplot in R (4.0.2) was used to draw the histogram (mean ± standard error (SE); SE = SD/sqrt (n)), cor to draw the correlation analysis chart (Zhao et al., 2022), and gplots and pheatmap to draw the heat map after the FPKM values of gene expression in three time periods were standardized.

## 3 Analysis of results

### 3.1 Physiological response dynamics of lateral branching of Pepino under different light qualities

#### 3.1.1 Effect of different light qualities on the plant height of Pepino seedlings

**Figure 2:**
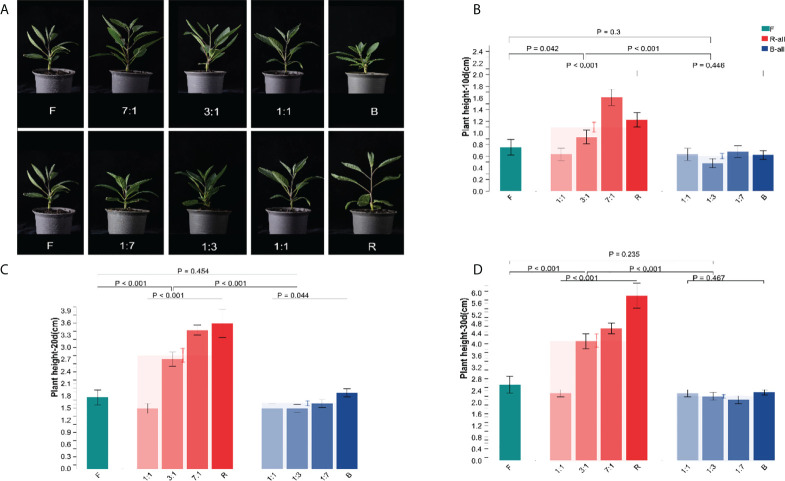
Effect of different light conditions on the height of Pepino seedlings. **(A)** the seedling phenotype of Pepino under different light treatments; **(B)** seedling height of Pepino under different light treatments for 10 d; **(C)** seedling height of Pepino under different light treatments for 20 d; **(D)** seedling height of Pepino under different light treatments for 30 d.

After 30 d of various light quality treatments, the growth of the seedlings height of Pepino was considerably different (**Figure 2A**). The plant height was reduced under blue light (B) treatment. Plant height growth under red light (R) treatment was significantly higher than that under full spectrum lamp (F). After 10 d of light quality treatment (**Figure 2B**), the plant height of the B-all treatment group was lower than that of the F treatment group. However, there was no significant difference in plant height between the same F and B-ALL treatment groups (*p* = 0.3). In contrast, after 10 d, the plant height of the R-all treatment group increased, and the seedlings treated with R and RB 7:1 showed significant differences (*p* = 0.042) compared with F. Meanwhile, RB 3:1 and RB 1:1 showed fewer differences than F. There was a significant difference between R-all and B-all (*p* < 0.001). After 20 d of light treatment (**Figure 2C**), there was a significant difference in plant height among treatments in the B-all group (*p* = 0.044). At 30 d (**Figure 2D**), the plant height of Pepino seedlings under R-all treatment was significantly higher than that of F and B (*p* < 0.001). These results indicated that Pepino was significantly responsive to red and blue light, and the plant height increased significantly with increasing R ratio, indicating that R promoted the growth of Pepino seedlings. However, the plant height decreased more significantly with an increased ratio of B, indicating that B inhibited the growth of Pepino seedlings. There was no significant difference between RB 1:1 and F groups.

#### 3.1.2 Effects of different light qualities on the lateral branches of Pepino seedlings

**Figure 3:**
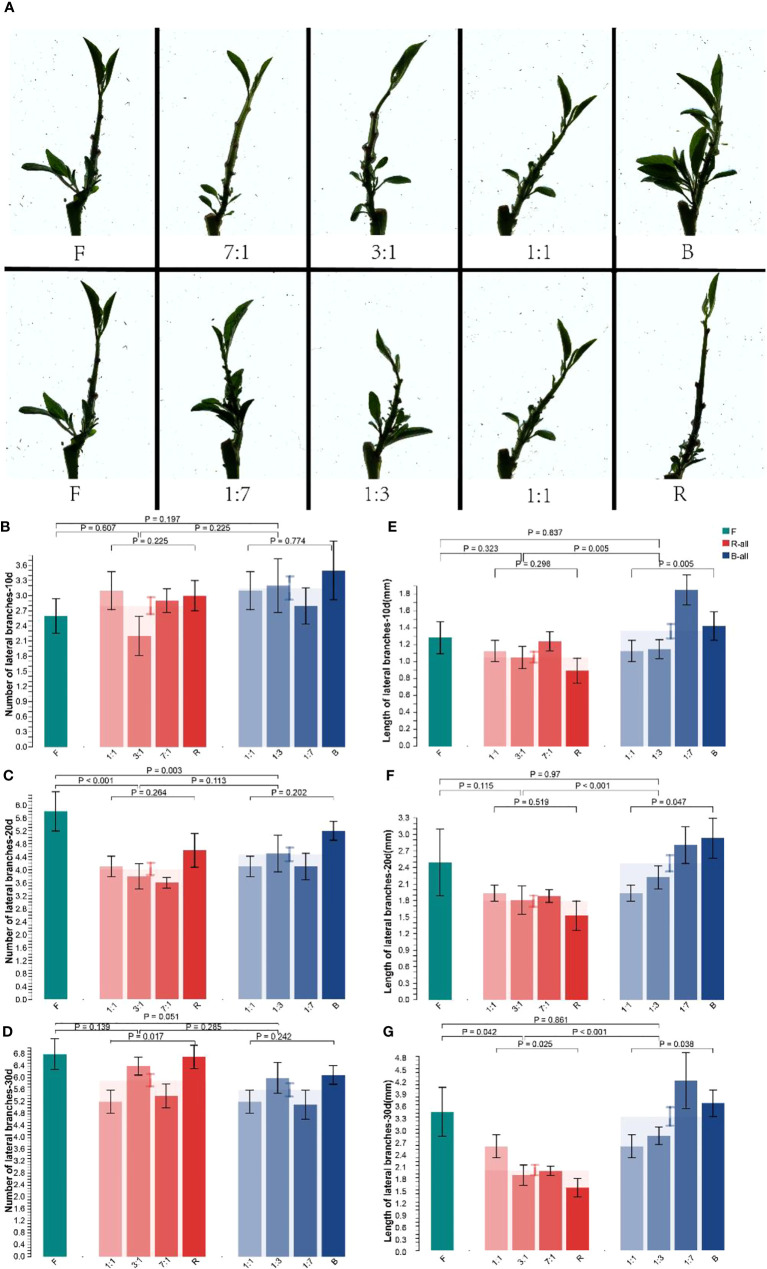
Effect of different light conditions on the lateral branching of Pepino seedlings. **(A)** seedling phenotype of Pepino under different light treatments; **(B)** number of lateral branches of seedlings of Pepino under different light treatments for 10 d; **(C)** number of lateral branches of seedlings of Pepino under different light treatments for 20 d; **(D)** number of lateral branches of seedlings of Pepino under different light treatments for 30 d; **(E)** length of lateral branches of Pepino under different light treatments for 10 d; **(F)** length of lateral branches of Pepino under different light treatments for 20 d; **(G)** length of lateral branches of Pepino under different light treatments for 30 d.

After the treatments, significant differences were observed in the lateral branches of the seedlings (**Figure 3A**). Compared with the F group, the number and length of lateral branches in the B group grew better after 30 d, and that in the R group was significantly inhibited. To clarify the effect of R and B treatments on lateral branches, we analyzed the increase in the number of lateral branches at different treatment times (**Figure 3B**). After 10 d of light quality treatment, the number of lateral branches among and within the F, R-all, and B-all groups did not increase significantly. However, the number of lateral branches under B treatment was significantly higher than that under F. After 20 d (**Figure 3C**), the increase in lateral branches in the R-all treatment group was significantly lower than that in the F treatment group (*p* < 0.001). R treatment inhibited the development of lateral branches of Pepino; however, there was no significant difference between B-all and F groups. After 30 d (**Figure 3D**), the differences among F, R-all, and B-all were not significant, and the inhibitory effect of R on the number of lateral branches was eliminated. The plant height growth in the B group was inhibited in the late stage; however, the increase in the plant height in the R group provided sufficient space for the growth of lateral branches, eliminating the difference in the number of lateral branches in the late stage.

Different light quality treatments not only significantly affect the number of lateral branches but also their length. After 10 d of light quality treatment, the length of lateral branches in B-all increased significantly (*p* = 0.005) compared with R-all; however, the length of lateral branches in RB 1:1 and RB 1:3 did not increase significantly (**Figure 3E**). After 30 d (**Figure 3G**), the length of lateral branches of the R-all group decreased significantly compared with that of F (*p* = 0. 042) and decreased more with increasing R treatment. The length of lateral branches of B-all and R-all groups increased significantly (*p* < 0.001). B treatment promoted the growth of lateral branches, whereas R treatment inhibited it. Overall, the effect of R and F treatments on plant height increased apical dominance, and the lateral meristem differentiation was partly inhibited, in contrast with the B treatment. Moreover, both plant height and length of lateral branches were significantly altered under R treatment compared with F treatment, indicating that the response of seedlings to R was high in the development process. Altogether, the lateral branches of the seedlings germinated vigorously, the plants were short, and the phenotypic changes under the B treatment were less evident than those under the F treatment.

### 3.2 Correlation analysis of physiological indexes of Pepino seedlings under different light qualities

**Figure 4:**
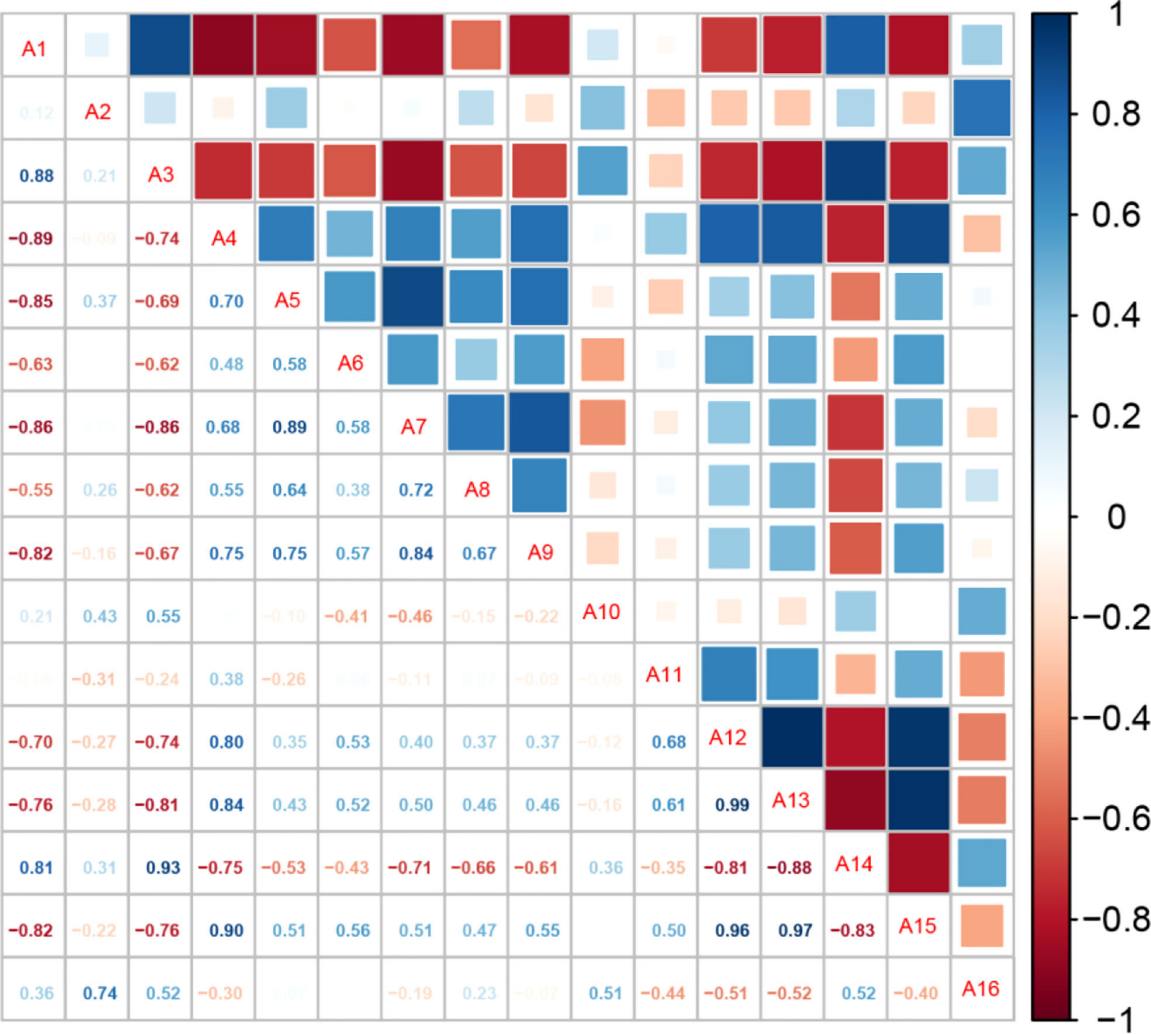
Correlation analysis among the indicators of Pepino under different light treatments. A1: percentage of blue light; A2: number of lateral branches; A3: length of lateral branches; A4: plant height; A5: aboveground dry weight; A6: number of leaves; A7: leaf area of the whole plant; A8: stem diameter; A9: internode length; A10: sucrose synthetase; A11: sucrose phosphate synthase; A12: Chl a; A13: Chl b; A14: Chl a/b; A15: carotenoids; A16: soluble sugar;.

To elucidate the correlation between the changes in lateral branches, plant height, and other physiological indexes, we conducted a correlation analysis of 16 physiological indexes according to the Pepino growth and development process regulated by different light environments (**Figure 4** and **Table S1-S2**). The length of lateral branches under different light quality treatments was significantly negatively correlated with plant height, aboveground dry weight, leaf area of the whole plant, internode length, and other aboveground indexes. The growth of lateral branches had antagonistic effects on plant height and internode length and competed with leaves for nutrients, resulting in decreased leaf area of the whole plant. The lateral branches were negatively correlated with Chl b and carotenoids and positively correlated with sucrose synthetase and Chl a/b. However, the relationship between plant height and each index contrasted with that of the length of lateral branches.

Further analysis of the correlation between the percentage of blue light and various indicators showed that the ratio of blue light was significantly positively correlated with the length of lateral branches and significantly negatively correlated with plant height. In addition, the ratio of blue light was significantly negatively correlated with aboveground dry weight, leaf number, total leaf area, internode length, Chl b, and carotenoids, and significantly positively correlated with Chl a/b, which was most consistent with the length of lateral branches. Blue light promoted the growth of the length of lateral branches and inhibited the production of carotenoids, resulting in a significant reduction in leaf area; however, the utilization rate of light energy was higher compared to other light treatments. However, red light facilitated the increase in plant height, internode length, total leaf area, and other indicators.

### 3.3 Gene expression profiles related to light response and lateral branching

**Figure 5:**
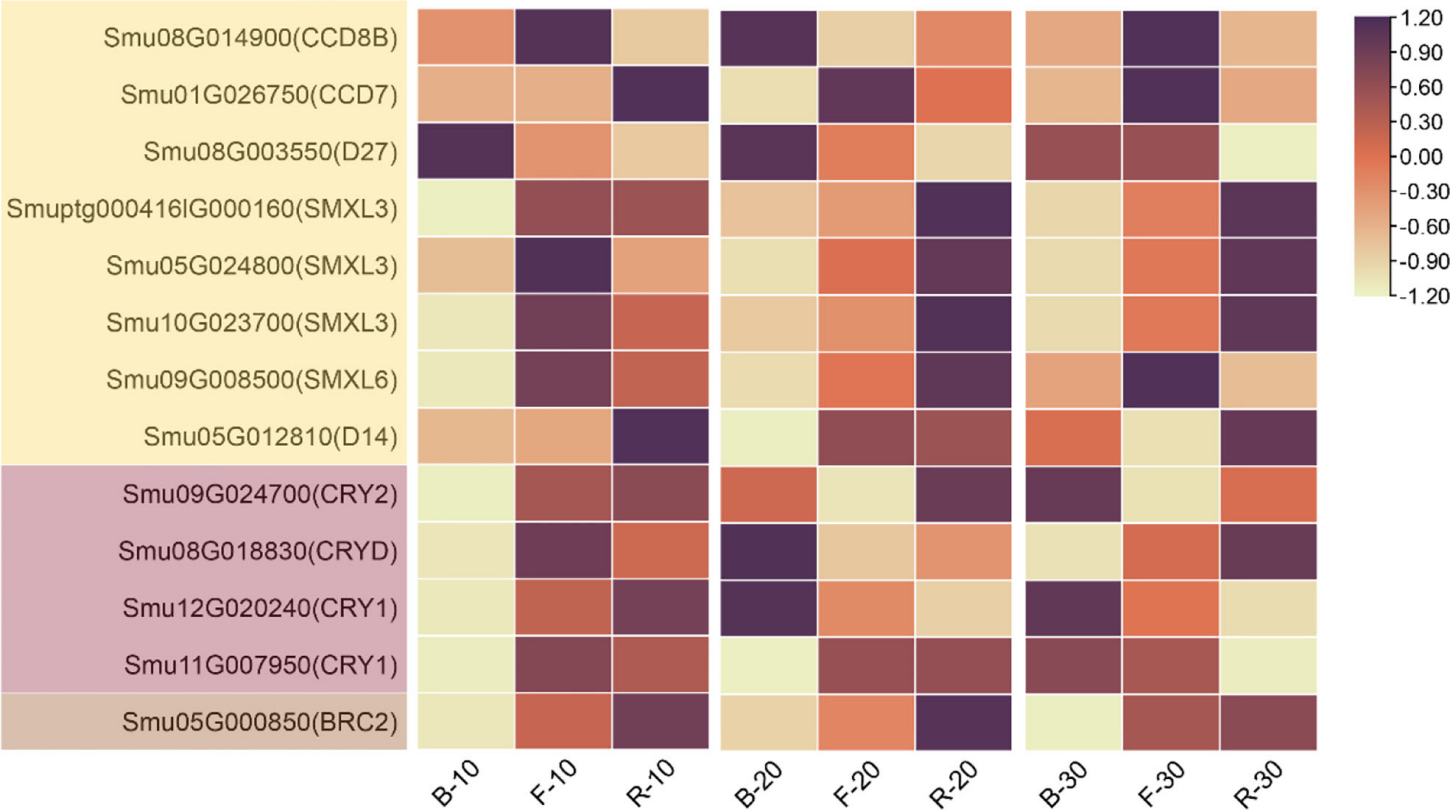
Heat map of *CCD*, *SMXL*, *D27*, *D14*, *CRY*, and *BRC2* expression under different periods of light quality treatments.

To elucidate the various regulatory mechanisms of lateral branching of Pepino and identify the genes responsible for lateral branching under different light quality treatments, we extracted the gene expression profiles related to light response and lateral branching from the transcriptome sequencing data, among which *CRY*, carotenoid cleavage dioxygenases (*CCD*), SMAX1-Like (*SMXL*), and dwarf14 (*D14*) were the most essential (**Figure 5**). After 10 d of light quality treatment, the expression specificity of strigolactone synthetic genes (β-carotenoid isomerase (*D27*), *CCD7*, *CCD8*) was low in each treatment. However, the expressions of strigolactone signal transduction genes (*SMXL*, *D14*), *CRY*, and *BRC2* were downregulated under B treatment. After 20 d, most genes related to strigolactone synthesis at the base of axillary buds were upregulated under B treatment, whereas genes related to signal transduction were upregulated under R treatment and downregulated under B treatment. Therefore, R did not promote strigolactone synthesis but promoted their signal transduction, thus promoting the expression of *BRC2* and inhibiting the lateral branching of plants under red light. On day 30, the relative content of strigolactone synthesis genes fluctuated, and their expression was high in F. The gene expression in strigolactone synthesis and signal transduction was low under B treatment, and *BRC2* expression was inhibited, resulting in significant elongation of lateral branches under B treatment. *CRY* expression increased gradually with the extension of light quality treatment time under blue light, indicating that *CRY* had a more significant response to blue light.

**Figure 6:**
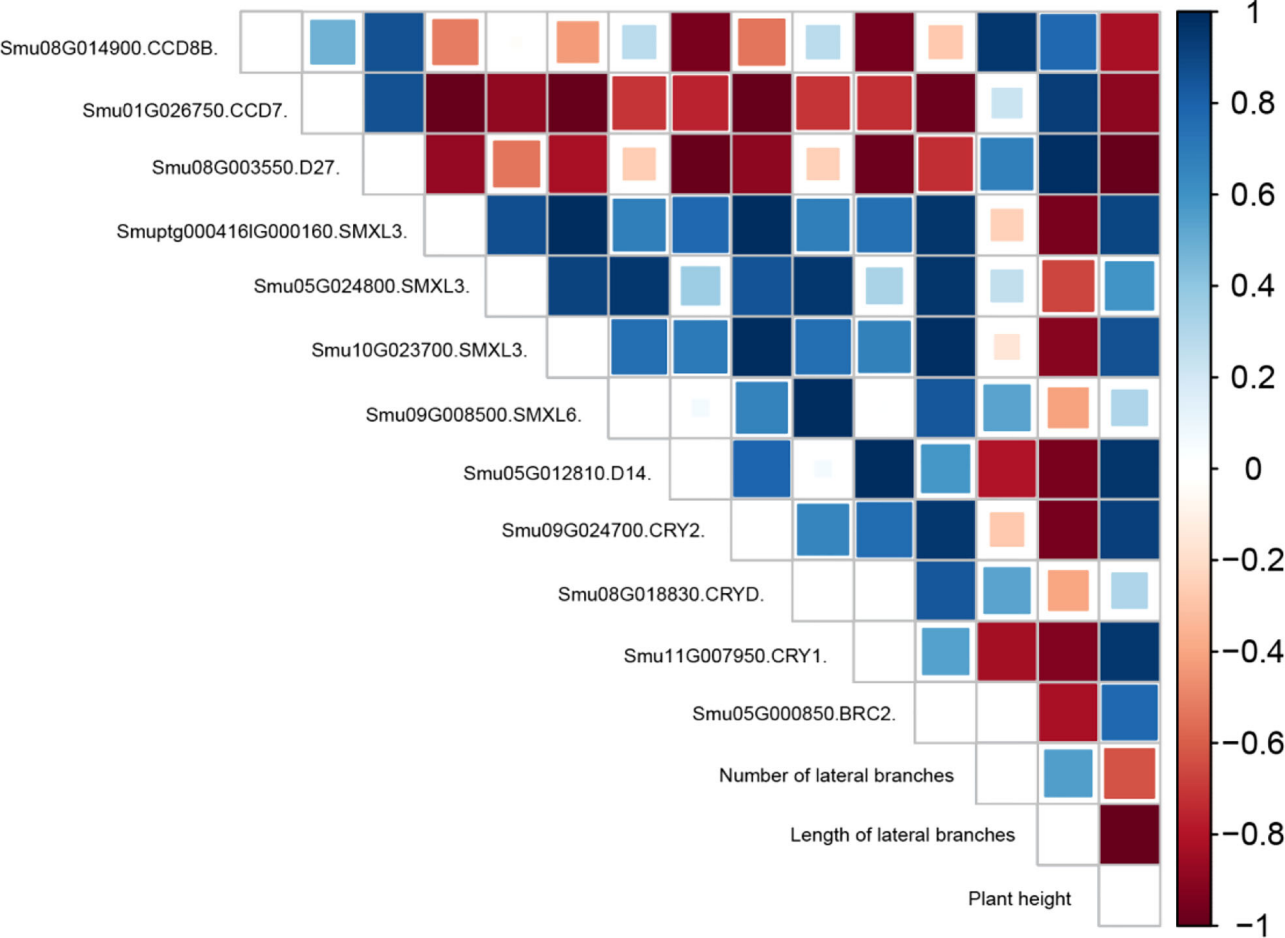
Correlation analysis between *CCD*, *SMXL*, *D27*, *D14*, *CRY*, and *BRC2* genes and physiological traits under different light quality treatments.

Based on the neglect of post-transcriptional translation regulation, the correlation analysis of *CCD*, *SMXL*, *D27*, *D14*, *CRY*, and *BRC2* genes and morphological indicators was conducted (**Figure 6** and **Table S3**). *SMXL* and *D14*, the key regulatory molecules of strigolactone signal transduction, were positively correlated with *BRC2* lateral branch regulatory factors and significantly negatively correlated with length and number of lateral branches. *D27*, *CCD7*, and *CCD8*, the key enzymes in strigolactone synthesis, were highly expressed under B treatment but negatively correlated with *BRC2*. Therefore, the synthesized strigolactones could not regulate the lateral branching of *BRC2* without signal transduction. In addition, *Smu09G024700* (*CRY2*) and *Smu11G007950* (*CRY1*) were negatively correlated with the length and number of lateral branches and positively correlated with the plant height.

## 4. Discussion

**Figure 7:**
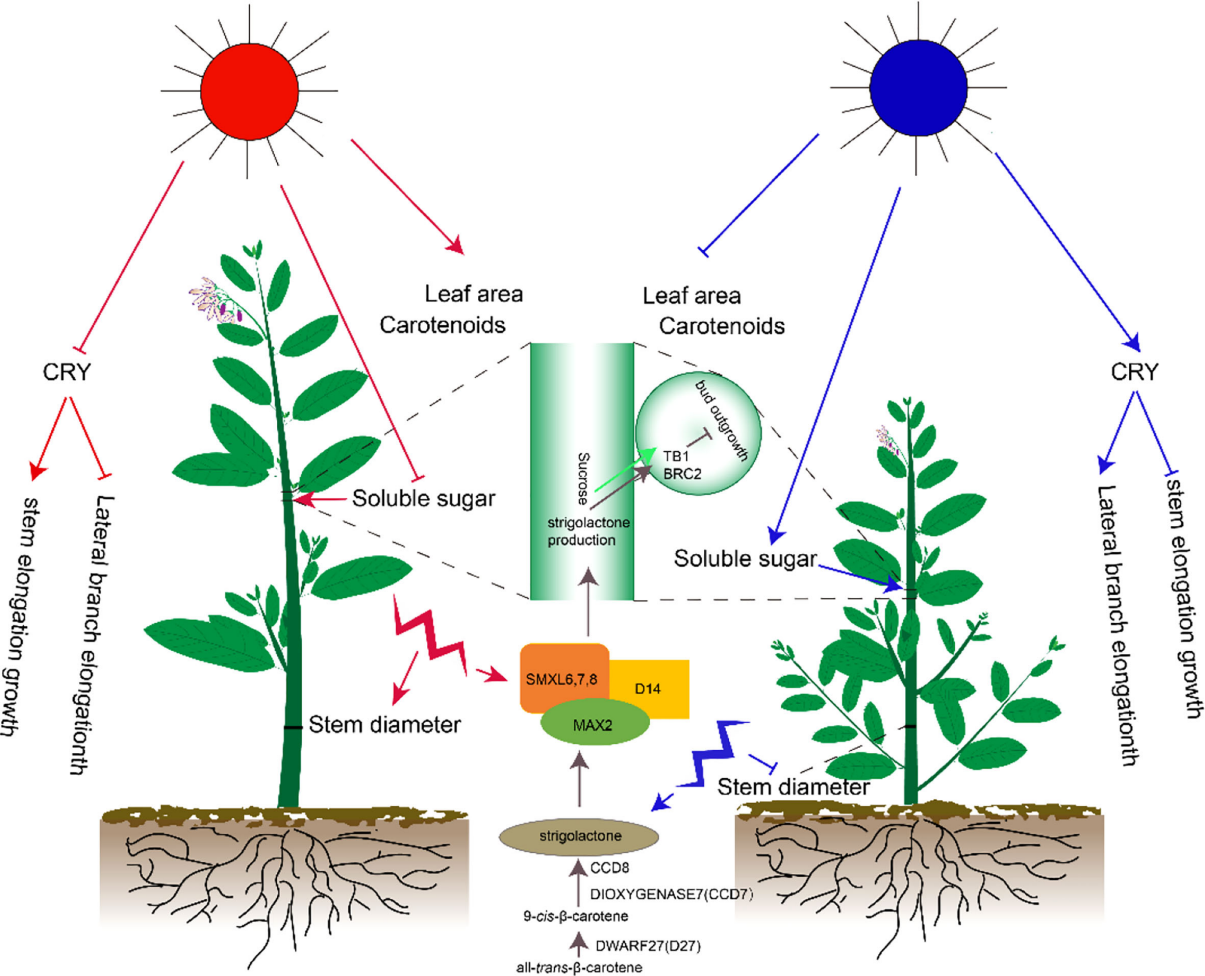
Effects of different light quality treatments on phenotypes, physiology, and gene expression of Pepino.

Different light qualities significantly affected the growth and development of Pepino. Red and blue lights played an important role in regulating the development of plants. In this study, the plant height, number of lateral branches, length of lateral branches, and other indexes of Pepino under different light quality treatments were compared to evaluate the different responses of lateral branches of Pepino to red and blue light (**Figure 7**). The response of plant height and lateral branches was one of the most important morphological indicators under red and blue light. In Petunia and other crops, B promoted elongation and reduced the number of lateral branches and biomass allocation of lateral branches (Miao et al., 2019). Under B treatment, phytochrome had no activity, resulting in internode elongation (Hoang et al., 2021) and plant height increase, which was inconsistent with the findings of our study. Our results showed that the plant height and internode length were significantly shortened under the B treatment, in contrast with the R treatment. B treatment promoted the length of lateral branches of Pepino but inhibited the plant height and internode elongation. We speculated that the response to red and blue light was species-specific. In the related studies of tomatoes, Arabidopsis, wheat, pepper, and other crops, the plant height increased significantly under monochromatic red light and decreased under monochromatic blue light (Liang et al., 2021). These researchers proposed that elongation was inhibited by activating *CRY* under blue light (Liu et al., 2016). *CRY* was highly expressed in the R group in the first 10 d of light quality treatment, whereas its expression was inhibited in the B group. However, the expression of *CRY* changed among treatments after 20 d; *CRY* was highly expressed in the B group, eliminating the height of B plants (Kong and Zheng, 2022), indicating that *CRY1* played a major role in mediating stem length inhibition (Ma et al., 2016). In addition, *CRY1* inhibits hypocotyl elongation by inhibiting BR (He et al., 2019) and gibberellin (GA) signaling (Yan et al., 2021). Therefore, the blue light-mediated a different mechanism of action of *CRY1*, resulting in the inhibition of the growth of lateral branches.

Strigolactones are a class of carotenoid-derived compounds (Waters et al., 2017) that trigger *MAX2* degradation by binding to the *D14* receptor and emitting signals through the interaction of inhibitory factors: *SMXL6*, *SMXL7*, and *SMXL8* (van Rongen et al., 2019). These SMXL inhibitory factors activate *BRC2* and inhibit the branching of axillary buds (Wang et al., 2020; Xie et al., 2020). In this study, *SMXL* and *D14* were highly expressed under R treatment, and *BRC2* gene expression was highly expressed under R treatment at all three periods. Therefore, we speculated that the inhibition of growth of axillary buds was mediated by *BRC1*. In addition, the specific expression of strigolactone synthesis genes, including *D27*, *CCD7*, and *CCD8*, under B, R, and F treatments were unclear. Hence, we speculated that different light treatments did not affect strigolactone synthesis but modulated *BRC2* expression by modulating strigolactone signaling, thus regulating the plant type of Pepino. Therefore, the different treatments of R and B caused significant differences in plant height and growth of lateral branches.

The change in the number of lateral branches under R and B treatments was not evident in this study. Under B treatment, the internodes were short, the plant height was restricted, and the increase in the number of lateral branches was not evident in the later stage. However, the inhibition of R on the number of lateral branches was also eliminated in the late stage because the increase in plant height impeded the production of more lateral leaves and longer internodes, increasing the number of lateral branches. The ratio of B and R light quality not only responded to morphological indicators such as plant height and lateral branches but also altered the leaf area and the contents of carotenoids, sucrose synthetase, Chl a, and Chl b. Correlation analysis revealed a significant negative correlation between the leaf area of the whole plant and the ratio of blue light but a positive correlation with the ratio of red light, indicating that the expansion of the leaf area was inhibited under blue light (Dougher and Bugbee, 2004; Nanya et al., 2012). Different branches would lead to different changes in leaf number and area (Rosenqvist et al.). Owing to the increase in the number of branches under the B group, the leaf number, leaf area, and stem diameter reduced due to the competition for nutrients. A high-intensity red light was associated with carotenoid accumulation and high oxygen absorption rate. When switched to blue light, carotenoid content would decrease significantly (Xu and Harvey, 2019), and the content of Chl b would negatively correlate with B (Vitale et al., 2020). In this study, the ratio of blue light was significantly negatively correlated with carotenoids, which was consistent with previous studies. An increase in chlorophyll and carotenoid content was observed under monochromatic red light.

Sucrose is a major regulator of bud growth, and artificially increasing plant sucrose levels inhibits the expression of branched1 (*BRC1*), thereby promoting the elongation of lateral buds (Xi et al., 2020). Our data supported the theory of apical dominance that the strong demand for sugars by the shoot tip inhibited the growth of axillary buds by limiting the amount of sugar transported to axillary buds (**Figure 7**). There was a significant positive correlation between the length of lateral branches and contents of sucrose synthetase and soluble sugar. The results showed that when the length of lateral branches was elongated, sucrose synthetase decomposed sucrose and provided energy for lateral branching.

In this study, the response of Pepino seedlings to different light qualities was elucidated by measuring the relevant morphological and physiological indicators. Our findings laid the foundation for understanding Pepino’s light quality response mechanism. Because the seedling development was very sensitive to red and blue wavelengths, an appropriately designed light formula might be an effective tool to improve Pepino’s plant phenotype and photosynthetic characteristics in a short time.

## 5. Conclusion

This study aimed to explore the lateral branching and plant height occurrence of Pepino seedlings using different light quality treatments. Through various mechanisms of action, blue light inhibited plant height elongation and promoted the growth and development of lateral branches of Pepino seedlings, in contrast with red light. The number of lateral branches showed no evident response under prolonged treatment (30 d). Furthermore, leaf area expanded, and plant dry weight increased under R treatment, whereas plant soluble sugar content increased under B treatment. Moreover, different light qualities regulated lateral branching by mediating different pathways such as strigolactone and *CRY*. Our findings helped elucidate the light quality response mechanism of Pepino and provided a theoretical reference for the regulation of lateral branching of Pepino by different light qualities.

## Data availability statement

The datasets presented in this study can be found in online repositories. The names of the repository/repositories and accession number(s) can be found below: National Center for Biotechnology Information (https://www.ncbi.nlm.nih.gov/bioproject/PRJNA869486; Accession to cite for these SRA data PRJNA869486).

## Author contributions

Writing—original draft preparation, CS, writing—review and editing, SY; methodology and investigation, XL; supervision, GZ; funding acquisition and conceptualization, QZ. All authors contributed to the article and approved the submitted version.

## Funding

Key Laboratory Project of Qinghai Science & Technology Department: 2020-ZJ-Y02, Qinghai Key Research and Development Conversion Project: 2022-NK-117, Qinghai Agriculture and Forestry Science Innovation Fund: 2021-NKY-02.

## Acknowledgments

We thank Bullet Edits Limited for linguistic editing and proofreading of the manuscript.

## Conflict of interest

The authors declare that the research was conducted in the absence of any commercial or financial relationships that could be construed as a potential conflict of interest.

## Publisher’s note

All claims expressed in this article are solely those of the authors and do not necessarily represent those of their affiliated organizations, or those of the publisher, the editors and the reviewers. Any product that may be evaluated in this article, or claim that may be made by its manufacturer, is not guaranteed or endorsed by the publisher.
