# Supplementary material for: Effects of light spectrum on the morphophysiology and gene expression of lateral branching in Pepino (Solanum muricatum)
Source: Front Plant Sci. 2022 Sep 23;13:1012086. doi: 10.3389/fpls.2022.1012086 (PMC9540516; doi:10.3389/fpls.2022.1012086)
Supplement: Supplementary file 1 [file DataSheet_1.docx]

Supplementary Material

Table S1 Determination of morphological indices of pepino under different light quality treatments

|  | A2 | A3 | A4 | A5 | A6 | A7 | A8 | A9 |
| --- | --- | --- | --- | --- | --- | --- | --- | --- |
| F-10d | 2.6±1.02 | 1.29±0.57 | 1.61±0.42 | 0.06±0.01 | 4.67±0.47 | 1654.6±157.32 | 1.46±0.13 | 0.29±0.03 |
| R-10d | 3±0.89 | 0.89±0.45 | 0.755±0.4 | 0.06±0 | 4.67±0.47 | 1970.3±136.01 | 1.5±0.07 | 0.26±0.04 |
| 7:1-10d | 2.9±0.7 | 1.24±0.34 | 1.24±0.34 | 0.05±0.01 | 5.33±1.25 | 1784.73±391.03 | 1.39±0.07 | 0.43±0.05 |
| 3:1-10d | 2.2±1.17 | 1±0.37 | 1±0.37 | 0.07±0.01 | 3.67±0.47 | 1739.77±90.89 | 1.5±0.07 | 0.4±0.04 |
| 1:1-10d | 3.1±1.14 | 1.13±0.37 | 1.13±0.37 | 0.04±0 | 4±0.82 | 1324.5±157.41 | 1.21±0.04 | 0.2±0.01 |
| 1:3-10d | 3.2±1.6 | 1.15±0.34 | 1.15±0.34 | 0.05±0.01 | 5.33±1.25 | 1278.73±232.78 | 1.42±0.23 | 0.13±0.02 |
| 1:7-10d | 2.8±1.08 | 1.85±0.54 | 0.68±0.3 | 0.03±0 | 4±0.82 | 867.77±40.58 | 1.2±0.06 | 0.11±0.01 |
| B-10d | 3.5±1.75 | 1.43±0.51 | 0.62±0.22 | 0.04±0.01 | 3.67±0.47 | 911.07±81.1 | 1.33±0.13 | 0.19±0.01 |
| F-20d | 5.8±0.98 | 2.49±1.18 | 3.43±0.37 | 0.11±0 | 7±0 | 3201±196.91 | 1.52±0.12 | 0.33±0.04 |
| R-20d | 4.6±1.56 | 1.53±0.8 | 1.77±0.55 | 0.13±0.01 | 7±0.82 | 3939.63±571.74 | 1.6±0.11 | 0.32±0.05 |
| 7:1-20d | 3.6±0.49 | 1.88±0.35 | 1.88±0.35 | 0.11±0.02 | 7.67±0.47 | 3808.57±469.44 | 1.48±0.19 | 0.58±0.05 |
| 3:1-20d | 3.8±1.17 | 1.8±0.73 | 1.8±0.73 | 0.1±0.02 | 7.33±0.47 | 4089.37±297.95 | 1.79±0.07 | 0.53±0.04 |
| 1:1-20d | 4.1±0.94 | 1.94±0.44 | 1.94±0.44 | 0.1±0.02 | 7.33±0.47 | 3244.57±388.25 | 1.46±0.12 | 0.3±0.06 |
| 1:3-20d | 4.5±1.69 | 2.22±0.63 | 2.22±0.63 | 0.1±0.01 | 8±0 | 2924±56.11 | 1.47±0.2 | 0.25±0.02 |
| 1:7-20d | 4.1±1.22 | 2.81±1 | 1.62±0.29 | 0.07±0.01 | 6.33±0.47 | 2068.23±206.03 | 1.23±0.08 | 0.18±0.02 |
| B-20d | 5.2±0.87 | 2.93±1.09 | 1.88±0.3 | 0.08±0.01 | 6.67±0.47 | 2784.67±60.4 | 1.4±0.15 | 0.21±0.02 |
| F-30d | 6.8±1.54 | 3.43±1.81 | 4.525±0.55 | 0.18±0.01 | 8.67±0.47 | 3975.2±333.39 | 1.76±0.11 | 0.52±0.07 |
| R-30d | 6.7±1.19 | 1.55±0.69 | 2.6±0.86 | 0.21±0.03 | 9.33±0.47 | 5116.93±467.14 | 1.76±0 | 0.58±0.11 |
| 7:1-30d | 5.4±1.2 | 1.97±0.33 | 1.97±0.33 | 0.17±0.01 | 9.33±0.94 | 4472.77±297.83 | 1.87±0.07 | 0.75±0.04 |
| 3:1-30d | 6.4±0.92 | 1.94±0.73 | 1.94±0.73 | 0.15±0.02 | 8.67±0.47 | 4930.9±725.95 | 1.99±0.11 | 0.67±0.05 |
| 1:1-30d | 5.2±1.17 | 2.58±0.84 | 2.58±0.84 | 0.15±0.03 | 9±0.82 | 4633.6±1017.5 | 1.8±0.04 | 0.48±0.1 |
| 1:3-30d | 6±1.55 | 2.84±0.64 | 2.84±0.64 | 0.1±0.02 | 8±1.41 | 3027.73±264.2 | 1.91±0.09 | 0.3±0.02 |
| 1:7-30d | 5.1±1.45 | 4.2±2.08 | 2.08±0.4 | 0.09±0.01 | 8.33±0.47 | 2387.37±108.48 | 1.65±0.19 | 0.25±0.02 |
| B-30d | 6.1±0.94 | 3.64±0.99 | 2.335±0.3 | 0.12±0.01 | 8.67±0.47 | 3400.07±180.44 | 1.87±0.09 | 0.28±0.01 |

Note: A2: number of lateral branches; A3: length of lateral branches; A4: plant height; A5: aboveground dry weight; A6: number of leaves; A7: leaf area of the whole plant; A8: stem diameter; A9: internode length;

Tables S2 Determination of physiological indexes of pepino under different light quality treatments

|  | A10 | A11 | A12 | A13 | A14 | A15 | A16 |
| --- | --- | --- | --- | --- | --- | --- | --- |
| F-10d | 920.51±39.89 | 458.38±50.77 | 1.26±0.11 | 0.36±0.03 | 3.49±0.02 | 0.23±0.02 | 0.01±0 |
| R-10d | 901.23±141.5 | 387.6±27.82 | 2.16±0.15 | 0.74±0.08 | 2.95±0.19 | 0.4±0.04 | 0.01±0 |
| 7:1-10d | 975.31±106.2 | 636.71±22.87 | 1.68±0.35 | 0.5±0.11 | 3.4±0.09 | 0.32±0.07 | 0.02±0 |
| 3:1-10d | 783.52±107.97 | 576.76±200.69 | 1.31±0.15 | 0.44±0.13 | 3.13±0.55 | 0.27±0.04 | 0.02±0 |
| 1:1-10d | 581.34±160.52 | 492.68±100.15 | 1.43±0.38 | 0.43±0.11 | 3.32±0.04 | 0.24±0.06 | 0.02±0 |
| 1:3-10d | 414.29±152.27 | 372.2±83.58 | 1.61±0.07 | 0.5±0.01 | 3.19±0.06 | 0.27±0.02 | 0.02±0 |
| 1:7-10d | 574.07±61.88 | 130.58±47.85 | 1.41±0.1 | 0.4±0.03 | 3.55±0.01 | 0.26±0.01 | 0.01±0 |
| B-10d | 637.7±136.56 | 398.57±89.44 | 1.17±0.02 | 0.38±0.05 | 3.15±0.48 | 0.19±0.01 | 0.01±0 |
| F-20d | 753.85±191.64 | 243.59±19.19 | 1.47±0.13 | 0.49±0.05 | 2.98±0.14 | 0.29±0.03 | 0.01±0 |
| R-20d | 684.62±230.85 | 1094.87±191.88 | 2.52±0.13 | 0.88±0.04 | 2.88±0.02 | 0.47±0.03 | 0.01±0 |
| 7:1-20d | 420.51±140.07 | 541.03±40.38 | 2.29±0.16 | 0.8±0.06 | 2.88±0.08 | 0.43±0.03 | 0.02±0 |
| 3:1-20d | 725.64±324.32 | 587.18±32.23 | 2.59±0.04 | 0.89±0 | 2.89±0.04 | 0.48±0.01 | 0.01±0 |
| 1:1-20d | 294.87±32.23 | 466.67±178.13 | 1.85±0.23 | 0.61±0.08 | 3.01±0.03 | 0.33±0.04 | 0.02±0 |
| 1:3-20d | 382.05±26.15 | 801.28±182.64 | 2.1±0.07 | 0.68±0.03 | 3.07±0.03 | 0.36±0.01 | 0.01±0 |
| 1:7-20d | 928.21±45.44 | 1000.96±280.97 | 1.96±0.06 | 0.64±0.02 | 3.07±0.03 | 0.34±0.01 | 0.01±0 |
| B-20d | 641.03±224.82 | 1053.53±126.56 | 1.93±0.15 | 0.58±0.03 | 3.35±0.08 | 0.33±0.03 | 0.02±0 |
| F-30d | 927.46±180.08 | 410.01±33.46 | 1.33±0.11 | 0.42±0.04 | 3.19±0.09 | 0.24±0.02 | 4.35±0.17 |
| R-30d | 689±176.75 | 402.54±70.22 | 1.31±0.07 | 0.44±0.02 | 2.97±0.05 | 0.26±0.01 | 1.64±0.03 |
| 7:1-30d | 610.26±186.99 | 696.2±50.45 | 1.47±0.19 | 0.51±0.06 | 2.9±0.06 | 0.29±0.04 | 2.24±0.09 |
| 3:1-30d | 542.38±129.5 | 446.33±40.98 | 1.05±0.09 | 0.38±0.03 | 2.76±0.12 | 0.2±0.02 | 2.33±0.21 |
| 1:1-30d | 674.36±206.53 | 428.13±118.28 | 1.08±0.05 | 0.36±0.02 | 3.05±0.05 | 0.19±0.01 | 1.68±0.17 |
| 1:3-30d | 1144.13±151.45 | 616.84±165.04 | 1.3±0.06 | 0.43±0.02 | 3.05±0.01 | 0.25±0.01 | 3.37±0.02 |
| 1:7-30d | 1493.39±377.48 | 495.76±60.02 | 1.16±0.09 | 0.38±0.04 | 3.06±0.11 | 0.22±0.01 | 2.07±0.37 |
| B-30d | 1061.94±234.96 | 614.41±45.37 | 1.21±0.13 | 0.37±0.04 | 3.27±0.15 | 0.2±0.03 | 3.11±0.44 |

Note: A10: sucrose synthetase; A11: sucrose phosphate synthase; A12: Chl a; A13: Chl b; A14: Chl a/b; A15: carotenoids; A16: soluble sugar;

Tables S3 CCD, SMXL, D27, D14, CRY, and BRC2 expression under different periods of light quality treatments（FPKM）

|  | B-10 | F-10 | R-10 | B-20 | F-20 | R-20 | B-30 | F-30 | R-30 |
| --- | --- | --- | --- | --- | --- | --- | --- | --- | --- |
| Smu08G014900(CCD8B) | 11.45 | 16.16 | 9.76 | 3.46 | 0.73 | 1.62 | 0.51 | 3.64 | 0.27 |
| Smu01G026750(CCD7) | 0.01 | 0.01 | 0.03 | 0 | 0.04 | 0.02 | 0 | 0.7 | 0.05 |
| Smu08G003550(D27) | 25.06 | 11.13 | 6.22 | 27.34 | 14.84 | 6.21 | 6.95 | 6.95 | 5.1 |
| Smuptg000416lG000160(SMXL3) | 8.72 | 9.99 | 9.95 | 2.88 | 3.41 | 5.63 | 3.5 | 4.67 | 6.41 |
| Smu05G024800(SMXL3) | 1.46 | 1.95 | 1.53 | 0.75 | 1.2 | 1.62 | 0.72 | 0.77 | 0.83 |
| Smu10G023700(SMXL3) | 2.13 | 3.27 | 2.87 | 1.96 | 2.72 | 4.83 | 1.77 | 2.6 | 3.63 |
| Smu09G008500(SMXL6) | 25.95 | 58.06 | 47.81 | 26.91 | 27.99 | 29.21 | 44.5 | 45.36 | 44.37 |
| Smu05G012810(D14) | 0.25 | 0.35 | 1.42 | 0.19 | 0.39 | 0.38 | 2.31 | 1.6 | 2.93 |
| Smu12G020240(CRY1) | 5.7 | 10.65 | 12.89 | 3.34 | 1.87 | 1.17 | 6.21 | 3.94 | 1.83 |
| Smu09G024700(CRY2) | 37.25 | 58.47 | 61.18 | 14.94 | 12.7 | 16.42 | 51.8 | 41.62 | 47.19 |
| Smu08G018830(CRYD) | 0.9 | 2.22 | 1.72 | 5.73 | 5.12 | 5.27 | 3.16 | 3.33 | 3.46 |
| Smu11G007950(CRY1) | 49.06 | 85.56 | 79.16 | 34.29 | 37.77 | 37.82 | 88.94 | 88.14 | 83.14 |
| Smu05G000850(BRC2) | 1.81 | 2.55 | 2.94 | 1.53 | 1.86 | 2.46 | 1.23 | 2.4 | 2.56 |
